# The evolution of hard tick-borne relapsing fever borreliae is correlated with vector species rather than geographical distance

**DOI:** 10.1186/s12862-021-01838-1

**Published:** 2021-05-31

**Authors:** Ranna Nakao, Kentaro Kasama, Bazartseren Boldbaatar, Yoshitoshi Ogura, Hiroki Kawabata, Atsushi Toyoda, Tetsuya Hayashi, Ai Takano, Ken Maeda

**Affiliations:** 1 Department of Veterinary Medicine, Joint Faculty of Veterinary Medicine, Yamaguchi University, 1677-1 Yoshida, Yamaguchi City, Yamaguchi 753-8515 Japan; 2 Department of Bacteriology, Faculty of Medical Sciences, Kyushu University, 3-1-1 Maidashi, Higashi-ku, Fukuoka City, Fukuoka 812-8582 Japan; 3 Laboratory of Virology, Institute of Veterinary Medicine, Mongolian University of Life Sciences, Zaisan, 17024 Ulaanbaatar, Mongolia; 4 Division of Microbiology, Department of Infectious Medicine, Kurume University School of Medicine, 67 Asahi-machi, Kurume City, Fukuoka 830-0011 Japan; 5 Department of Bacteriology-I, National Institute of Infectious Disease, 1-23-1 Toyama, Shinjyuku-ku, Tokyo, 162-8640 Japan; 6 Department of Genomics and Evolutionary Biology, National Institute of Genetics, 1111 Yata, Mishima City, Shizuoka 411-8540 Japan; 7 Joint Graduate School of Veterinary Medicine, Yamaguchi University, 1677-1 Yoshida, Yamaguchi, 753-8515 Japan; 8 Department of Veterinary Science, National Institute of Infectious Diseases, 1-23-1 Toyama, Shinjyuku-ku, Tokyo, 162-8640 Japan

**Keywords:** Hard-tick borne relapsing fever, *Borrelia miyamotoi*, Whole-genome, Evolution

## Abstract

**Background:**

Relapsing fever (RF) borreliae are arthropod-borne spirochetes and some of them cause human diseases, which are characterized by relapsing or recurring episodes of fever. Recently, it has been classified into two groups: soft tick-borne RF (STRF) borreliae and hard tick-borne RF (HTRF) borreliae. STRF borreliae include classical RF agents and HTRF borreliae, the latter of which include *B. miyamotoi*, a human pathogen recently identified in Eurasia and North America.

**Results:**

In this study, we determined the genome sequences of 16 HTRF borreliae strains: 15 *B. miyamotoi* strains (9 from Hokkaido Island, Japan, 3 from Honshu Island, Japan, and 3 from Mongolia) and a *Borrelia* sp. tHM16w. Chromosomal gene synteny was highly conserved among the HTRF strains sequenced in this study, even though they were isolated from different geographic regions and different tick species. Phylogenetic analysis based on core gene sequences revealed that HTRF and STRF borreliae are clearly distinguishable, with each forming a monophyletic group in the RF borreliae lineage. Moreover, the evolutionary relationships of RF borreliae are consistent with the biological and ecological features of each RF borreliae sublineage and can explain the unique characteristics of *Borrelia anserina*. In addition, the pairwise genetic distances between HTRF borreliae strains were well correlated with those of vector species rather than with the geographical distances between strain isolation sites. This result suggests that the genetic diversification of HTRF borreliae is attributed to the speciation of vector ticks and that this relationship might be required for efficient transmission of HTRF borreliae within vector ticks.

**Conclusions:**

The results of the present study, together with those from previous investigations, support the hypothesis that the common ancestor of borreliae was transmitted by hard-bodied ticks and that only STRF borreliae switched to using soft-bodied ticks as a vector, which was followed by the emergence of *Borrelia recurrentis*, lice-borne RF borreliae. Our study clarifies the phylogenetic relationships between RF borreliae, and the data obtained will contribute to a better understanding of the evolutionary history of RF borreliae.

**Supplementary Information:**

The online version contains supplementary material available at 10.1186/s12862-021-01838-1.

## Background

*Borrelia* are spiral-shaped bacteria (spirochetes) that are transmitted primarily by ticks or lice, and some *Borrelia* species cause Lyme disease (LD) or relapsing fever (RF) in humans. At present, borreliae are classified into three phylogenetic groups: LD borreliae, RF borreliae, and reptile-associated (REP) borreliae [16, 55]. The vectors of LD borreliae and REP borreliae are hard-bodied ticks (e.g., *Ixodes* spp., *Amblyomma* spp., and *Hyalomma* spp.). RF borreliae have long been thought to be transmitted by soft-bodied ticks (e.g., *Ornithodoros* spp. or *Argas* spp.), except for *Borrelia recurrentis* and *B. theileri*, which are transmitted by human body lice (*Pediculus humanus*) and hard-bodied ticks (*Rhipicephalus* spp.), respectively [3, 53]. However, in 1995, Fukunaga and coworkers isolated a novel species of RF borreliae named *Borrelia miyamotoi* from hard-bodied ticks, *Ixodes persulcatus*, and wild rodents, *Apodemus argenteus*, on Hokkaido Island, Japan [17]. Since then, several novel species of RF borreliae have been found in hard-bodied ticks: *Borrelia lonestari* from *Amblyomma americanum*, *Borrelia* sp. AGRF from *Amblyomma geoemydae*, *Borrelia* sp. BR from *Rhipicephalus microplus*, and *Borrelia* sp. tHM16w from *Haemaphysalis* spp. [2, 18, 57, 62]. Furthermore, the first human cases of *B. miyamotoi* infection were reported in Russia in 2011, followed by cases in the USA, Europe, and Japan [21, 23, 44, 49]. Recent characterization of these hard-bodied tick-borne relapsing fever (HTRF) borreliae revealed that HTRF borreliae are (1) phylogenetically related to traditional RF borreliae, (2) transmitted by hard-bodied ticks, (3) detected with a low prevalence from field-collected ticks, and (4) difficult to cultivate using media for borrelia cultivation [4, 10, 60]. In addition, it has been found that *B. miyamotoi* is transmitted transovarially in vector ticks and develops a high level of bacteremia in field-collected mice [4, 48].

It is known that most *Borrelia* species are transmitted by specific vectors and are related to hard-bodied ticks, except soft-bodied tick-borne relapsing fever (STRF) borreliae and *B. recurrentis*. Therefore, it was recently suggested that a host-switching event may have occurred involving the ancestor of STRF borreliae [55]. However, in phylogenetic analyses based on housekeeping genes, HTRF borreliae formed a monophyletic group within STRF borreliae (Additional file 3: Figure S1) [28, 52, 55]. The same finding was obtained by phylogenetic analysis based on the chromosomal sequence of *B. miyamotoi* LB-2001 [5]. This discrepancy between biological and ecological features (e.g., vector species) and phylogenetic relationships was partially resolved by whole-genome sequence comparison of REP borreliae; STRF borreliae and HTRF borreliae diverged from a common ancestor of RF borreliae [19]. In this analysis, however, *B. anserina*, which is thought to be the ancestral species of STRF borreliae [14], was not clearly separated from other STRF borreliae.

*B. miyamotoi* is distributed across the Northern Hemisphere, and several genotypes have been identified in species of *Ixodes* ticks, mainly in the *Ixodes ricinus* complex [30]. In the USA, American type *B. miyamotoi*, sequence type (ST) 634 and ST683, was found in *I. scapularis* and *I. pacificus*, respectively. While, the European type (ST635) was found in *I. ricinus* in western Europe, and the Siberian type (ST633 and ST680) was found in *I. persulcatus* in eastern Europe, Russia and Asia. Although, ST633 was found from eastern Europe to Hokkaido, Japan, ST680 has been only found from Honshu island, Japan. In Japan, in addition to the Siberian type, a novel genotype (ST682) of *B. miyamotoi* was isolated from *I. ovatus*, which is genetically distinct from the *I. ricinus* complex [27]. Moreover, a novel *Borrelia* sp. tentatively named tHM16w (ST735) was isolated from *Haemaphysalis megaspinosa* in Japan [18]. In previous genome-wide analyses [24, 29, 32, 33], *B. miyamotoi* strains isolated in limited geographic areas (USA, Russia and Netherlands) and from limited tick species (only those in the *I. ricinus* complex, e.g., *I. ricinus*, *I. scapularis* and *I. pacificus*) or human patients were analyzed due to the difficulty of the detection and cultivation of *B. miyamotoi*. In this study, we determined the genome sequences of several genotypes of *B. miyamotoi* isolated from *I. persulcatus*, *I. pavlovskyi* and *I. ovatus* in Japan and Mongolia and of *Borrelia* sp. tHM16w, a close relative of *B. miyamotoi*, and compared these sequences with those of previously sequenced *B. miyamotoi* as well as representative strains from a wide range of borreliae to understand the evolution of HTRF borreliae and their adaptation to tick vectors.

## Results

### Genome sequencing of HTRF borreliae

**Fig. 1:**
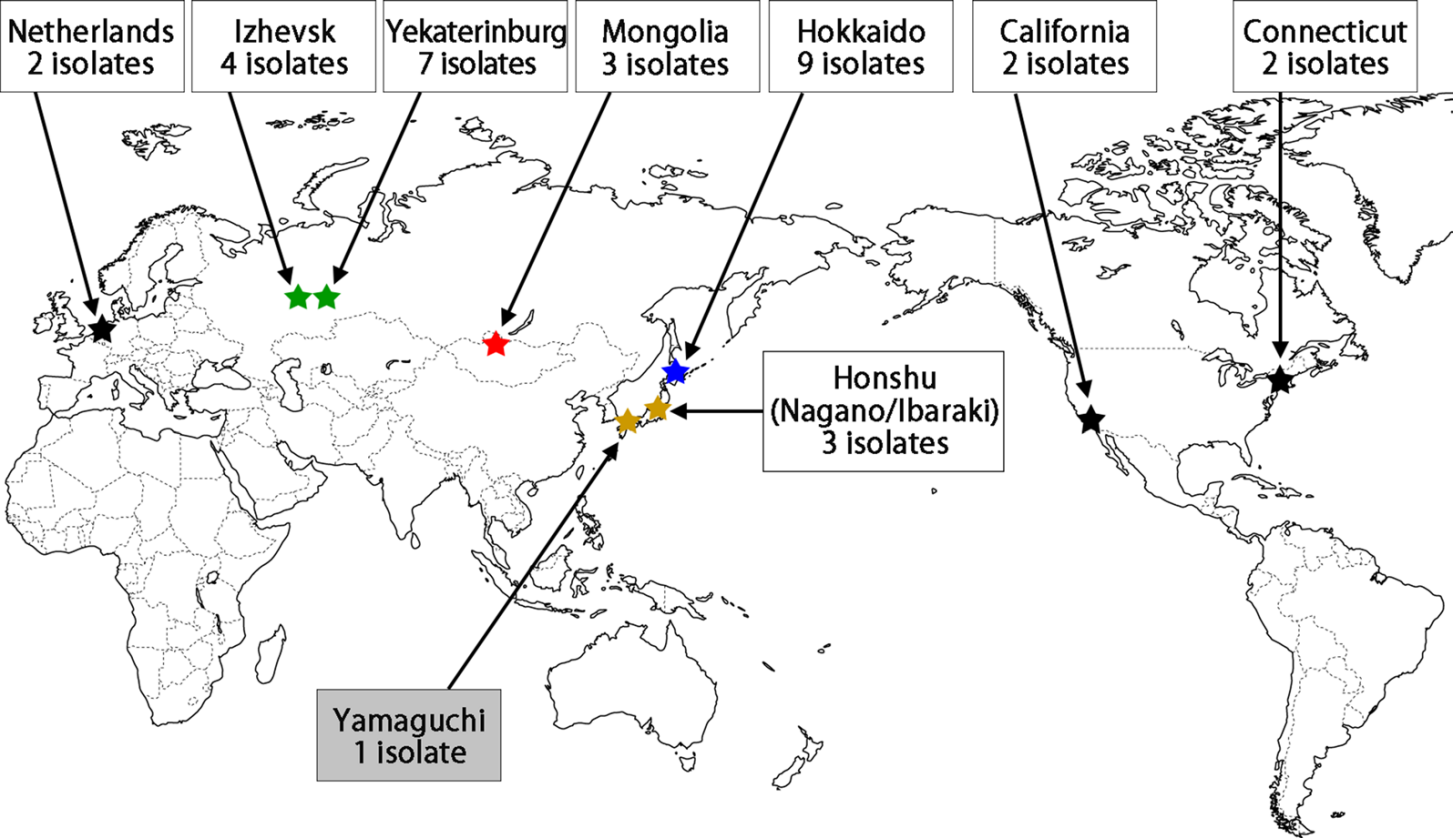
Geographic information on the isolation sites of *B. miyamotoi* strains analyzed in this study. The isolation site of *Borrelia* sp. tHM16w is also indicated (gray box)

In this study, we sequenced 15 *B. miyamotoi* strains isolated in Japan and Mongolia. The *Borrelia* sp. tHM16w clone 2-D isolated from *H. megaspinosa* in Japan was also sequenced (Fig. 1 and Table 1).

**Table 1:** Strains sequenced in this study

Species	Strain	Sequencer	Region	Year	Isolation source	ST	chDNA general features					Accession No
							Length	GC %	CDS	tRNA	rRNA	
*B. miyamotoi*	HT31^T^	MiSeq	Hokkaido, Japan	1990	*Ixodes persulcatus*	633	9,05,850	28.87	827	32	3	AP024371
	HT24	MiSeq	Hokkaido, Japan	1990	*I. persulcatus*	633	9,06,376	28.87	831	32	3	AP024372
	HK004	MiSeq	Hokkaido, Japan	1993	*I. persulcatus*	633	9,05,693	28.87	829	32	3	AP024373
	NB103/1	MiSeq	Hokkaido, Japan	1991	*I. persulcatus*	633	9,06,055	28.87	829	32	3	AP024374
	MYK1 clone G3	PacBio + MiSeq	Hokkaido, Japan	2012	*Ixodes pavlovskyi*	633	9,05,196	28.87	862	32	3	AP024375
	MYK2	MiSeq	Hokkaido, Japan	2012	*I. persulcatus*	633	9,06,441	28.87	831	32	3	AP024391
	MYK3	MiSeq	Hokkaido, Japan	2012	*I. persulcatus*	633	9,05,982	28.88	829	32	3	AP024392
	MYK4	MiSeq	Hokkaido, Japan	2012	*I. persulcatus*	633	9,05,872	28.88	826	32	3	AP024393
	MYK5	MiSeq	Hokkaido, Japan	2012	*I. persulcatus*	633	9,06,016	28.88	828	32	3	AP024394
	M12C4	MiSeq	Bulgan, Mongolia	2014	*I. persulcatus*	633	9,06,281	28.87	866	32	3	AP024395
	M15A8 clone C7	MiSeq	Selenge, Mongolia	2014	*I. persulcatus*	633	9,05,679	28.87	831	32	3	AP024396
	M20E6	MiSeq	Khuvsgul, Mongolia	2015	*I. persulcatus*	633	9,06,111	28.87	832	32	3	AP024397
	Y14T1	MiSeq	Nagano, Japan	2014	*I. persulcatus*	680	9,05,843	28.88	832	32	3	AP024398
	Y15T1 clone 13	MiSeq	Nagano, Japan	2015	*I. persulcatus*	680	9,05,723	28.88	833	32	3	AP024399
	Y14T18	PacBio + HiSeq	Ibaraki, Japan	2014	*Ixodes ovatus*	682	9,04,737	28.85	822	32	3	AP024400
*Borrelia* sp.	tHM16w clone 2-D	PacBio + HiSeq	Yamaguchi, Japan	2016	*Haemaphysalis megaspinosa*	735	9,14,768	27.73	815	32	3	AP024401

In this study, two *B. miyamotoi* strains and *Borrelia* sp. tHM16w clone 2-D were sequenced by PacBio/MiSeq/HiSeq. Remaining 13 *B. miyamotoi* strains were sequenced by MiSeq only. Although, the genome quality was differed between these strains, nearly complete linear chromosome sequences were obtained for each strain by gap filling (Table 1 and Additional file 2: Table S1). The 15 *B. miyamotoi* strains belonged to ST633 (n = 12), ST680 (n = 2) or ST682 (n = 1), and *Borrelia* sp. tHM16w clone 2-D belonged to ST735. The general features of the chromosomes of these strains are summarized in Table 1.

The chromosomes of the sequenced *B. miyamotoi* strains were approximately 905 kb (904,737–906,441 bp) in length and had a GC content of 28.9%, consistent with the chromosomes of previously sequenced *B. miyamotoi* strains (Additional file 2: Table S1) [24, 29, 32, 33]. The chromosome of *Borrelia* sp. tHM16w clone 2-D was 914,768 bp in length with a GC content of 27.7%. Pulsed-field gel electrophoresis (PFGE) analysis of two selected *B. miyamotoi* strains and *Borrelia* sp. tHM16w clone 2-D showed that, unlike STRF borreliae, these HTRF borreliae harbored 2 or 3 large plasmids and several small plasmids (Additional file 4: Figure S2). As the plasmid sequences obtained were highly fragmented due to the presence of various multicopy cassettes encoding membrane proteins [5, 11], plasmids were excluded from further analysis. The chromosomes of *B. miyamotoi* strains were highly conserved despite the differences in their geographical origins and tick host species (Additional file 5: Figure S3). On each chromosome, 822–866 protein-coding sequences (CDSs) were identified. Among these, 816 were conserved in all 15 *B. miyamotoi* strains, and most of the strain-specific CDSs were apparently degraded (split or truncated) (data not shown). Chromosome synteny was also well conserved between *B. miyamotoi* and *Borrelia* sp. tHM16w 2-D, and 786 (96%) of the 816 core CDSs of *B. miyamotoi* were conserved in *Borrelia* sp. tHM16w 2-D. Four CDSs (BmHA_00210, encoding a tetratricopeptide repeat-containing protein; BmHA_00402 and BmHA_00563, encoding hypothetical proteins; and BmHA_00703, encoding a ferritin-like protein; CDS numbers were from the ST633 strain MYK1 G3) showed a high level of variation in intragenic tandem repeat regions among the ST633 and ST680 *B. miyamotoi* strains sequenced in this study (Additional file 6: Figure S4).

### Phylogenetic relationship among HTRF and STRF borreliae inferred based on the core gene sequences

**Fig. 2:**
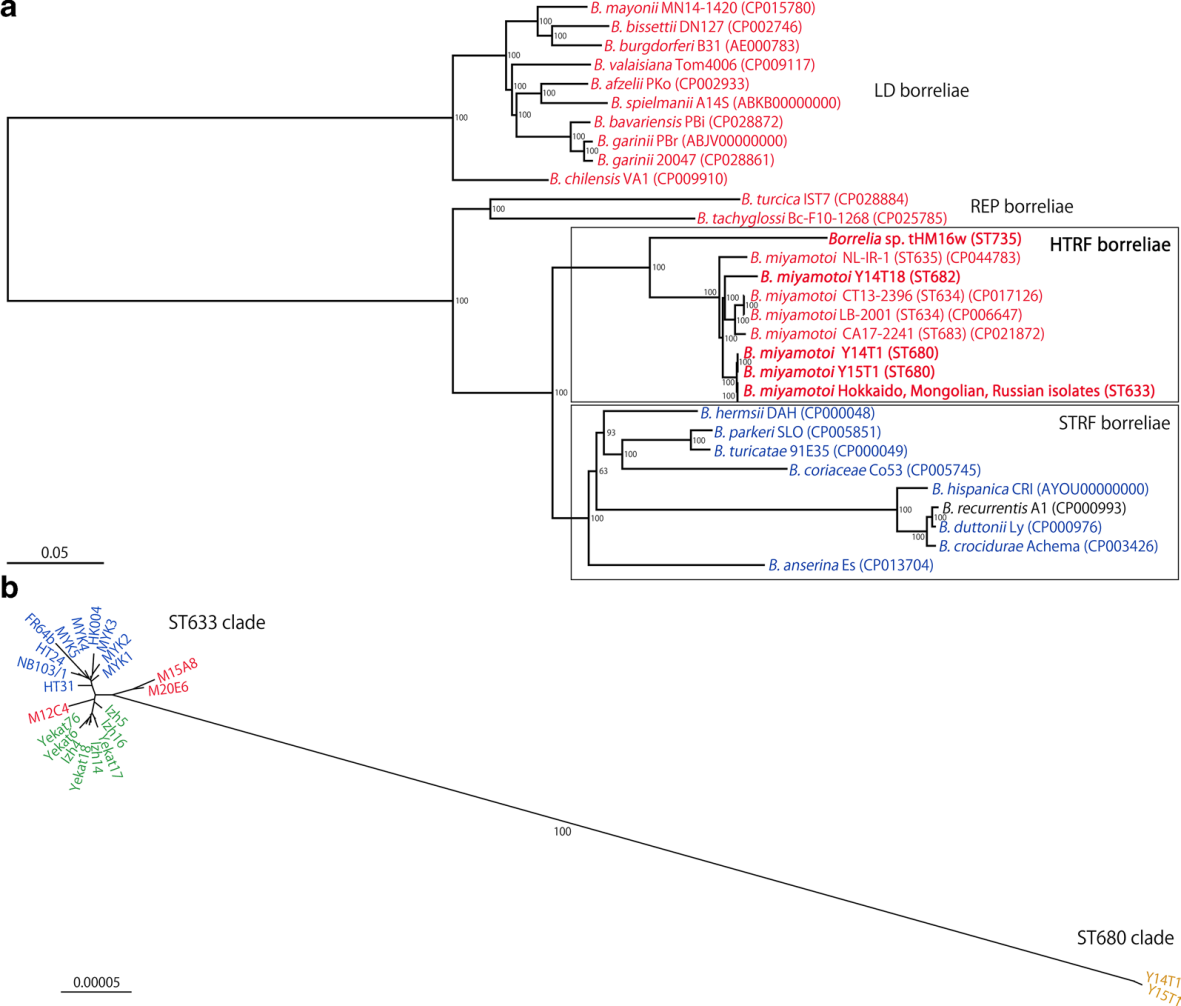
Phylogenetic trees of the entire borrelia lineage and ST633/ST680 based on the core chromosomal gene. **A** Based on the sequences of 567 core chromosomal genes of the entire borrelia lineage, a maximum-likelihood (ML) phylogenetic tree was constructed using RAxML with 1000 bootstrap replicates. Only bootstrap values over 60% are indicated. The bacteria transmitted by hard ticks, soft ticks, and lice are indicated in red, blue, and black, respectively. The sequence accession number and ST of each HTRF borreliae strain are shown in parentheses. The ST633 branch includes 21 sequences (10 Hokkaido isolates, 3 Mongolian isolates, and 8 Russian isolates). Sequences obtained in this study are indicated in bold. The scale bar indicates 5% sequence divergence. **B** An unrooted ML tree based on the sequences of 567 core chromosomal genes of 21 ST633 and 2 ST680 strains. Ten ST633 Hokkaido isolates, 8 ST633 Russian isolates, 3 ST633 Mongolian isolates, and 2 ST680 strains (both isolated in Honshu, Japan) are indicated in blue, green, red and yellow, respectively. The scale bar indicates 0.005% divergence

We performed phylogenetic analysis of the entire borrelia lineage based on the sequences of core chromosomal genes (n = 567) to understand the phylogenetic position of HTRF in borrelia and the relationship between HTRF and STRF borreliae. In this analysis, LD borreliae was used as outgroup of RF borreliae, because the root of borreliae was located between LD and common ancestor of RF and REP borreliae by phylogenetic analysis based on house-keeping gene (Additional file 3: Figure S1). The phylogenetic analysis based on core genes revealed that LD, REP, and RF borreliae formed distinct clades and that HTRF and STRF borreliae formed a monophyletic group in the RF borreliae lineage (Fig. 2A). Importantly, the results indicate that *B. anserina*, which has been thought to be the ancestral species of STRF borreliae, was the first species to separate from other STRF borreliae in the STRF lineage. In the HTRF lineage, *Borrelia* sp. tHM16w 2-D was clearly separated from *B. miyamotoi* strains, and *B. miyamotoi* strains belonging to ST633 and ST680 were clustered together to form a subclade distinct from the strains belonging to other STs. The ST682 strain sequenced in this study, which was isolated from *I. ovatus* in Japan (Honshu), was not clustered with the ST633/680 strains. Similar analysis of *B. miyamotoi* ST633/680 strains based on their core chromosomal gene sequences clearly separated ST633 and ST680 strains and revealed that the root of ST633 was located between the subclade comprising the two Mongolian isolates and the root of the group containing all the other ST633 strains, including all Hokkaido and Russian isolates and one Mongolian isolate (Fig. 2B).

### Comparison of ANI values

The average nucleotide identity based on the BLAST algorithm (ANIb) values between *B. miyamotoi* strains (calculated using chromosome sequences; Additional file 2: Table S2) were over 96.31%, which is higher than the cutoff value (95%) generally used for species delineation [20]. While the pairwise ANIb values among ST633/ST680 strains (ST633, isolated mainly from *I. persulcatus* in Hokkaido, Mongolia, and Russia; ST680, isolated from *I. persulcatus* in Honshu) were over 99.86%, those between the ST633/ST680 strains and the ST682 strain (isolated from *I. ovatus* in Honshu) ranged from 97.13 to 97.16%. Interestingly, the ANIb values between the ST633/ST680 strains and the ST634 strains (isolated from *I. scapularis* in the USA) ranged from 97.79 to 97.81%, and those between the ST633/ST680 strains and the ST635 strains (isolated from *I. ricinus* in the Netherlands) also ranged from 97.30 to 97.33%. These results suggested that the ST633/ST680 strains isolated in Japan are genetically more closely related to ST634 strains isolated in the USA than to the ST682 strain isolated in Japan. Moreover, although the ST633 strains were isolated in a wide range of geographical areas from Asia to Russia, their chromosomal sequences were highly conserved, as indicated by ANIb values over 99.97%.

### Phylogenetic relationships among ST633 strains

**Fig. 3:**
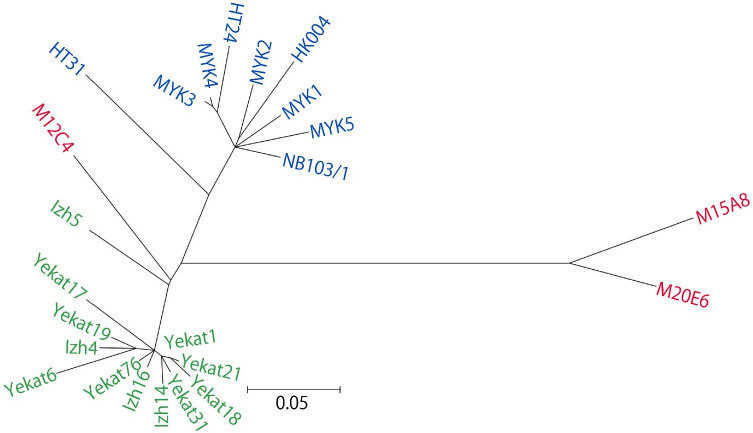
An unrooted phylogenetic tree of ST633 strains based on the SNPs identified in entire chromosomes. Hokkaido (n = 9), Russian (n = 12), and Mongolian isolates (n = 3) are indicated in blue, green, and red, respectively. The sequences of Russian isolates were obtained from the GenBank database. The scale bar indicates 5% divergence. The ML tree was constructed based on 207 SNPs identified in whole chromosomes using MEGA7

To understand the more precise phylogenetic relationships among ST633 strains, we analyzed the single nucleotide polymorphisms (SNPs) across the entire chromosome sequence in 24 ST633 strains. This analysis identified only 207 SNPs in total, although these strains were isolated in Japan (Hokkaido), Mongolia and Russia (Additional file 2: Table S3). Phylogenetic analysis based on the identified SNPs showed that the strains from Hokkaido and Russia formed monophyletic clades (Fig. 3). Although two Mongolian isolates formed a clade distinct from the Hokkaido and Russian clades, one Mongolian isolate, strain M12C4, belonged to the Russian clade. Pairwise SNP distances within the Hokkaido and Russian clades ranged from 2 to 34 and from 1 to 35 SNPs, respectively. It should be noted that although 4 of the 9 Hokkaido isolates were isolated in the early 1990s (1990–1993) and 5 were isolated in 2012, no time-dependent subclustering was observed for these strains. Intriguingly, the pairwise SNP distances between the Hokkaido and Russian clades ranged from 30 to 45 SNPs, and those between the Hokkaido clade and the M15A8 and M20E6 Mongolian strains ranged from 53 to 67 SNPs. Because the core gene-based analysis of ST633/680 strains showed that the root of ST633 was located between the M15A8/M20E6 clade and the root of the Hokkaido and Russian clades (Fig. 2B), the result of the whole-chromosome SNP-based analysis indicates that the genetic distances among the three ST633 clades were not correlated with their geographic relationships.

### Correlation of the pairwise genetic distances of HTRF borreliae and vector tick species

**Fig. 4:**
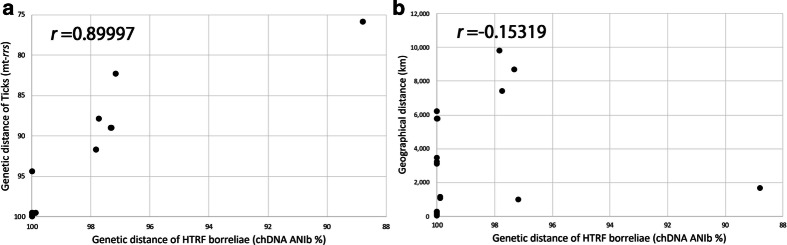
Comparison of the pairwise genetic distances of HTRF borreliae and vector ticks or geographical distance. Correlation of the pairwise genetic distances of HTRF borreliae and the mt-*rrs* of vector ticks (**A**) or geographical distance between isolation sites (**B**) were shown. The pairwise genetic distances of 29 HTRF borreliae strains and vector ticks were defined by chromosome ANIb values and mt-*rrs* sequence divergence, respectively, and geographical distances were calculated with Google Maps. Pearson’s correlation coefficients are indicated

Finally, we investigated how the genetic diversity of HTRF borreliae is correlated with their vector tick species or the geographical distances of their isolation sites. For this purpose, the pairwise genetic distances of HTRF borrelia strains were defined by the ANIb values of the chromosomal sequences, and the pairwise genetic distances of vector tick species and the pairwise geographical distances of their isolation sites were calculated based on the tick mitochondrial 16S rRNA gene (mt-*rrs*) sequence divergence and in Google Maps, respectively. Then, the correlation of the pairwise genetic distances of HTRF borrelia strains with those of vector ticks and of the geographical distances were calculated (Fig. 4A, B). In this analysis, the Pearson’s correlation coefficient between the pairwise genetic distances of HTRF borreliae and vector ticks was *r* = 0.89997 (*P* < 0.001), while that between the pairwise genetic distances and geographical distances of HTRF borrelia strains was *r* = − 0.15319 (*P* = 0.428). This result indicates that the genetic distance between HTRF borrelia strains is highly correlated with those of the vector species but not with the geographical distances between strain isolation sites.

## Discussion

In this study, we determined nearly complete chromosomal sequences of 16 HTRF strains. The general features of the determined chromosome sequences, such as genome size, GC content and genome architecture, were very similar to those of previously sequenced *B. miyamotoi* strains isolated in the USA, Russia, and the Netherlands (Table 1 and Additional file 2: Table S1) [13, 37]. In addition, chromosomal gene synteny was highly conserved among the HTRF strains sequenced in this study, even though they were isolated in different geographic regions and from different tick species (Additional file 5: Figure S3). Similar results were obtained in the comparison with previously sequenced *B. miyamotoi* strains isolated in other regions and from other tick species (data not shown).

There has been some ambiguity in the evolutionary relationship of HTRF borreliae with other *Borrelia* species because of the inconsistency between the reported phylogenetic relationships and biological differences between them [28, 52, 55]. However, in the phylogenetic analysis performed in this study based on core gene sequences, HTRF and STRF borreliae each formed a monophyletic group in the RF borreliae lineage (Fig. 2A). This phylogenetic relationship is consistent with the biological features of each group of borreliae; that is, LD, REP and HTRF borreliae are transmitted by hard-bodied ticks, and only STRF borreliae are transmitted by soft-bodied ticks or lice. Thus, our data support the hypothesis that a host-switching event occurred in the ancestor of STRF borreliae [55]. In our analysis, the African STRF borreliae (*B. duttonii*, *B. recurrentis* and *B. crocidurae*; the vectors of *B. recurrentis* are lice) and American STRF borreliae (*B. hermsii* and *B. turicatae*) groups were clearly separated, as previously reported [13]. In addition, the phylogenetic position of *B. anserina*, a causative agent of avian spirochetosis, in the STRF borreliae lineage was clarified. Since *B. anserina* has unique characteristics in terms of its host range (only birds), vector ticks (*Argas* sp.), and metabolic pathways, it has been considered distinct from other STRF borreliae [14]. However, in previous phylogenetic studies, *B. anserina* was not clearly separated from other STRF borreliae [5, 19]. In contrast, our analysis which included various *B. miyamotoi* strains and a *Borrelia* sp. tHM16w clone 2-D strain showed high confidence (bootstrap value; 100) that *B. anserina* was the first species to separate from other STRF borreliae, forming a unique sublineage in the STRF lineage (Fig. 2A). It seems that this phylogenetic position of *B. anserina* explains its unique biological properties which have been acquired during the long-time evolution.

Another interesting finding is the clear correlation of the genetic distances between HTRF borreliae strains with those of their vector tick species rather than with the geographical distances between strain isolation sites (Fig. 4A, B). This result suggests that the genetic diversification of HTRF borreliae is attributed to the speciation of their vector ticks. During the past two decades, many examples of the coevolution of symbiotic bacteria and their hosts, e.g., insects, plants or vertebrates, have been reported (reviewed by Gupta et al. [22]), including a recent report by Coimbra-Dores et al. [9] in which the authors observed co-cladogenesis between *Coxiella*-like endosymbiont strains and the lineages of their *Rhipicephalus* vector ticks. Because these symbiotic bacteria and their hosts usually include nutritional and metabolic collaboration, a close evolutionary association has occurred. In the case of parasites(pathogens)–hosts interactions, it was suggested that parasites diversification was affected by the geographical isolation. For instance, genetic diversity of *Plasmodium vivax* was significantly associated with geographic distance [15]. Borreliae may not be symbiotic bacteria, but most are transmitted by one or a few specific tick species [3, 38]. In fact, several studies have demonstrated vector competence between *Ixodes* ticks and LD borreliae (reviewed by Eisen et al. [12]). Vector competence, the ability of an arthropod to acquire, maintain, and successfully transmit a microorganism, is thought to be attributed to differences in the biological and ecological features of vector ticks and microorganisms and blood-feeding hosts [35, 41, 50]. Recently, the vector competence of geographical populations of *Ornithodoros turicata* ticks for the STRF borreliae species *B. turicatae* has been reported. While two different populations of bacteria similarly colonized in the salivary glands of ticks, the transmission efficiencies of these bacteria differed among various geographical populations of ticks [31]. The transmission efficiencies of HTRF borreliae within vector tick populations have not yet been investigated because of the difficulty of their cultivation and the low prevalence of questing vector ticks. However, the clear correlation of the pairwise genetic distances between HTRF borrelia strains with those between vector tick species observed in the present study suggests efficient transmission of HTRF borreliae in each vector tick species. On the other hand, this analysis included only two species; *B. miyamotoi* and *Borrelia* sp. tHM16w, a single outlier point in Fig. 4. Therefore, further investigation of other HTRF borreliae species are required for better understanding of diversification and evolution of HTRF borreliae.

The phylogenetic analysis based on SNPs of ST633 strains suggests a sign of geographic separation of the ST633 populations (Fig. 3). However, one Mongolian isolate belonged to the Russian clade. In addition, ST680 strains, which are the closest relatives of ST633 strains (Fig. 2B), were isolated in Honshu, Japan (Fig. 1). Thus, the genetic relationships of the ST633/680 strains analyzed in this study were not fully correlated with their geographic relationship. Separation of ST633 strains into three clades may simply represent local expansion of ST633 sublineages. *I. persulcatus* and *I. pavlovskyi*, the vector ticks of ST633 strains, infest not only mammalian hosts but also birds [27, 58]. *B. miyamotoi* was detected in bird-associated *I. persulcatus* and *I. ricinus* ticks in Japan and Sweden, respectively [17, 61]. Therefore, the wide geographic distribution of ST633 strains may be explained by the migration of birds, as proposed for LD borreliae [26, 39].

In the whole-chromosome SNP analysis of ST633 strains, we identified only 207 SNP sites among 24 strains isolated in Japan, Mongolia, and Russia (Additional file 2: Table S3), which accounted for just 0.02% of the chromosome sequence (207/905,196 bp). Although, the strains have been cultivated by artificial medium, we sequenced low passage strains. Therefore, the small number of SNPs suggests that their chromosomes are highly conserved despite the notable difference in isolation year and geographical region. A similar clonal structure was reported in *Rickettsia japonica*, a causative agent of tick-borne Japanese spotted fever in humans [1]. The frequency of SNPs in the *R. japonica* genome was reported to be 0.009% (112/1,283,227–1,284,037 bp) within strains from various regions in Japan. Both *B. miyamotoi* and *R. japonica* are transovarially transmitted in ticks [4, 48]. Therefore, transovarial transmission in vector ticks might be one of the bottleneck in the diversification of chromosome sequences of tick-borne bacteria.

## Conclusions

Our present analysis shows that HTRF and STRF borreliae are phylogenetically clearly distinguishable, and each forms a monophyletic clade in the RF borreliae lineage. The evolutionary relationship of RF borreliae inferred by our analysis is consistent with the biological and ecological features of each RF borreliae sublineage and can explain the unique features of *B. anserina*. Thus, the results of the present study, together with those of previous investigations [56, 57], provide strong support for the hypothesis that the common ancestor of borreliae was transmitted by hard-bodied ticks and that only STRF borreliae switched to using soft-bodied ticks as a vector; this was followed by the emergence of *B. recurrentis,* which has adapted to lice. In addition, the pairwise genetic distance between HTRF borreliae strains was well correlated with vector species rather than geographical site of isolation, indicating that the genetic diversification of HTRF borrelia is attributable to the speciation of vector ticks.

## Methods

### Bacterial strains and culture conditions

We analyzed low passage of 15 strains of *B. miyamotoi* and a strain of *Borrelia* sp. tHM16w (Table 1). The ST of each strain was referred for multi locus sequence typing database (https://pubmlst.org/borrelia/). Of the 15 *B. miyamotoi* isolates, 12 belonged to ST633, two belonged to ST680, and one belonged to ST682. Four of the 15 strains were isolated in the 1990s in Hokkaido, and the others were isolated in the 2000s: 5 in Hokkaido, 3 on Honshu Island and 3 in Mongolia [17, 27, 59]. While 13 strains were isolated from *I. persulcatus*, one was from *I. pavlovskyi* and one was from *I. ovatus*. The *Borrelia* sp. tHM16w clone 2-D (ST735) was originally isolated from *H*. *megaspinosa* [18, 27]. Bacteria were cultivated at 30 °C using Barbour–Stoenner–Kelly-M medium for 3 or 4 weeks, and the growth of spirochetes was examined by dark-field microscopy (at a magnification of 200×) [58]. Cloning of strains was performed by limiting dilution cultivation in 96 deep well plate containing BSK-M medium (1.8 ml).

### DNA purification

Bacterial DNA for next-generation sequencing was prepared from cultured spirochetes using Genomic-tip 100/G (QIAGEN, Tokyo, Japan). The DNeasy Blood and Tissue Kit (QIAGEN), Wizard Genomic DNA Purification Kit and Wizard Plus SV Minipreps DNA Purification Kit (Promega Corporation, Madison, WI, USA) were also used to prepare DNA samples for gap filling.

### Genome sequencing

Two *B. miyamotoi* strains and the *Borrelia* sp. tHM16w clone 2-D was sequenced by the PacBio RS II system (Pacific Biosciences, CA, USA) followed by error correction using Illumina reads (Table 1). After assembling PacBio reads by HGAP3 [8], Illumina reads obtained with the MiSeq or HiSeq sequencer (Illumina Inc., CA, USA) were mapped. The details of sequencing and assembling were described in Additional file 1: Method and Additional file 2: Table S4.

Thirteen *B. miyamotoi* strains were sequenced using MiSeq and the paired-end sequence reads (300 bp × 2; 101,402–250,000 pair reads for each strain) were obtained. After assembling the illumina reads, scaffolds were mapped to assembled sequence of *B. miyamotoi* MYK1 G3 strain as the reference sequence. The number of reads and scaffolds of chDNA were shown in Additional file 2: Table S5.

### Genome finishing

Genome finishing was performed by capillary sequencing and Illumina read mapping. The capillary sequencing was performed for gaps which are likely due to the tandem repeat and some SNPs. PCR products were sequenced by an ABI3130XL sequencer (Applied Biosystems, CA, USA) or outsourcing at Eurofins Genomics Inc. (Tokyo, Japan). Illumina read mapping was performed for confirmation of SNPs and carried out using CLC Genomics Workbench ver. 7 (QIAGEN).

### Genome annotation and comparison

Genome sequences were autoannotated using Prokka ver. 1.12 with default parameters [51], and some CDSs were manually assigned by using IMC ver. 7.3.2 (In Silico Biology, Inc., Kanagawa, Japan). Comparison of chromosome sequences was performed using GenomeMatcher ver. 2.3 [42]. Tandem repeats were identified using Tandem Repeat Finder with default parameters [6]. The assembled sequences have been deposited in the DRA/SRA/ERA database under Bioproject and Biosample accession numbers; PRJDB10961 and SAMD00264446–SAMD00264461, respectively.

### Core gene and phylogenetic analyses of HTRF borreliae

Core chromosomal genes were determined for 29 HTRF borreliae strains, comprising 28 *B. miyamotoi* strains (10 Hokkaido strains, 3 Mongolian strains, 3 Honshu strains, 8 Russian strains, 3 USA strains, and one Netherland strain) and *Borrelia* sp. tHM16w clone 2-D (Table 1, Fig. 1, Additional file 2: Table S1), and the sequenced reference strains represented each of the other 21 species of borreliae. The sequences of reference strains were downloaded from the DDBJ/EMBL/GenBank database and used for the analysis after autoannotation using Prokka ver. 1.12 with default parameters. Core genes were identified by Roary [43] with a threshold of > 60% sequence identity, which was determined on the basis of the ANIb values between *B. duttonii* Ly and *B. burgdorferi* B31 (74.98%). A maximum likelihood (ML) tree based on the sequences of 567 genes identified as the core chromosomal genes of 50 strains of borreliae was constructed using RAxML ver. 8.0 [54] with the GTRGAMMA model and 1000 bootstrap replicates. The tree was visualized by Fig Tree ver. 1.4.4.

### Comparison of the genetic distance and geographical distance

ANIb values were calculated using JSpecies ver. 1.2.1 with default parameters [47]. The mt-*rrs* sequences were downloaded from the DDBJ/EMBL/GenBank database (see Additional file 2: Table S6 for accession numbers and geographic information for the tick collection sites) [7, 25, 27, 40, 45, 46, 58]. The mt-*rrs* sequences were aligned using ClustalW, and evolutionary divergences between species were calculated by the Kimura 2-parameter model using MEGA 7 [34]. Geographic distances were calculated using Google Maps (https://www.google.com/maps/). *B. miyamotoi* strain HT31 and *I. persulcatus* collected in Hokkaido were used as a reference for pairwise comparison of the genetic distance.

### Single nucleotide polymorphism (SNP) analysis of *B. miyamotoi* ST633 strains

For the SNP analysis of entire chromosomal sequences of *B. miyamotoi* ST633 strains (Table 1), both the strains from Hokkaido and Mongolia and Russian clinical isolates were analyzed. The sequence of MYK1 G3 was used as a reference for SNP calling, which was performed based on the alignment of complete sequences constructed using NUCmer with default parameters [36]. SNPs in tandem repeats were eliminated manually, and the 207 identified SNPs were aligned using ClustalW; the phylogenetic tree was constructed by the ML model in MEGA 7 [34].

## Supplementary Information

**Additional file 1.** Additional methods.

**Additional file 2: Table S1. **Reference strains of *B. miyamotoi* used in this study. **Table S2.** ANI values of chromosomes among HTRF borreliae. **Table S3.** SNPs identified in this study. **Table S4.** Summary information of NGS data. **Table S5.** Summary information of NGS data. **Table S6.** List of the tick mt-rrs sequences used in this study.

**Additional file 3: Figure S1.** Phylogenetic trees of the entire borrelia lineage based on DNA gyrase subunit B gene (*gyrB*) gene. The phylogenetic tree is constructed by the neighbour-joining method based on the Kimura 2-parameter model. The percentage of replicate trees in which the associated taxa clustered together in the bootstrap test (1000 replicates) is indicated next to the branches. Values < 70% have been omitted. The bar indicates the percentage of sequence divergence. The bacteria transmitted by hard ticks, soft ticks, and lice are indicated in red, blue, and black, respectively. Sequence of *B. anserina* is indicated in bold type. GenBank accession numbers of each strains are indicated.

**Additional file 4: Figure S2.** The plasmid repertoires of HTRF borreliae and STRF borreliae.

**Additional file 5: Figure S3.** Comparison of the whole-chromosome sequences of the HTRF borreliae sequenced in this study. The entire chromosomal sequences of five *B. miyamotoi* strains (HT31, MYK1 G3, M12C4, Y14T1, and Y14T18) and *Borrelia* sp. tHM16w 2-D were compared using GenomeMatcher ver. 2.3. Sequence identity is shown by heat map.

**Additional file 6: Figure S4.** Schematic presentation of intragenic tandem repeats found in four *B. miyamotoi* genes. Tandem repeats in BmHA_00210, BmHA_00402, BmHA_00563 and BmHA_00703 are schematically shown. The tandem repeats were identified using Tandem Repeat Finder. Each box indicates a repeat unit, and boxes with the same colors and patterns represent the same or very similar repeat sequences (less than 4 base differences from the consensus sequence).

## Data Availability

Public data used in this study are listed in Table 1 and Additional files. The datasets supporting the conclusions of this article are available in DRA/SRA/ERA database under Bioproject and Biosample Accession numbers; PRJDB10961 and SAMD00264446–SAMD00264461, respectively.

## Abbreviations

LD: Lyme disease
RF: Relapsing fever
REP: Reptile-associated
HTRF: Hard-bodied tick-borne relapsing fever
STRF: Soft-bodied tick-borne relapsing fever
ST: Sequence type
PFGE: Pulsed-field gel electrophoresis
CDSs: Protein-coding sequences
ANIb: Average nucleotide identity based on the BLAST algorithm
SNPs: Single nucleotide polymorphisms
mt-*rrs*: Tick mitochondrial 16S rRNA gene
