# Supplementary material for: The evolution of hard tick-borne relapsing fever borreliae is correlated with vector species rather than geographical distance
Source: BMC Ecol Evol. 2021 May 31;21:105. doi: 10.1186/s12862-021-01838-1 (PMC8166147; doi:10.1186/s12862-021-01838-1)
Supplement: Supplementary file 1 — Additional file 1. Additional methods. [file 12862_2021_1838_MOESM1_ESM.docx]

**Additional Methods**

*Genome sequencing and assembling*

PacBio sequencing libraries were prepared using the SMRTbell Template Prep Kit ver.1.0 (Pacific Biosciences), and run was done by P6-C4v2 chemistry with 360 min movies. After assembling PacBio reads by HGAP3 with default parameters (Chin et al., 2013), illumina reads obtained with the MiSeq or HiSeq sequencer (Illumina Inc., CA, USA) were mapped using the Burrows-Wheeler aligner for sequence-error correction with SAMtools ver.0.1.9 or Pilon with default parameters (Li and Durbin, 2009; Li et al., 2009; Walker et al., 2014).

Thirteen *B. miyamotoi* strains were sequenced using MiSeq. Sequencing libraries were prepared using the QIAseq FX DNA Library kit (QIAGEN), and paired-end sequence reads (300 bp x2; 101,402~250,000 pair reads for each strain) were obtained. The Illumina reads were trimmed by Pltanus_trim (<http://platanus.bio.titech.ac.jp/pltanus_trim>) and assembled using Platanus_B Assembler ver.1.1.0 (<http://platanus.bio.titech.ac.jp/platanus-b>) with default parameters (Kajitani et al., 2020). For mapping of scaffolds, we used assembled sequence of *B. miyamotoi* MYK1 G3 strain as the reference sequence of 13 *B. miyamotoi* strains.

*Genome finishing*

Genome finishing was performed by capillary sequencing of PCR products and Illumina read mapping. First, we performed capillary sequencing of gaps, which are likely due to the tandem repeat, and some SNPs. Then illumina reads were mapped to gap-filled sequences for curation. For capillary sequencing, primers were designed based on scaffolds sequences and PCR was performed using Tks Gflex DNA Polymerase or PrimeSTAR GXL DNA Polymerase (TaKaRa Bio Inc.). PCR products were purified with a High Pure PCR Product Purification Kit (Roche Diagnostics GmbH) or Wizard SV Gel and PCR Clean-Up System (Promega Corporation) and sequenced by an ABI3130XL sequencer (Applied Biosystems, CA, USA) in our laboratory or at Eurofins Genomics Inc. (Tokyo, Japan). Illumina read mapping was carried out using CLC Genomics Workbench ver. 7 (QIAGEN). Illumina reads were trimmed by removing the 5’-terminal 20 bp and 3’-terminal 30 bp, and reads shorter than 150 bp were discarded. Then, reads were mapped to the reference sequences with a threshold of >80% coverage and 100% (HiSeq reads) or 90% (MiSeq reads) mapping identities.

*Phylogenetic analyses based on DNA gyrase subunit B (*gyrB*) gene*

Sequences of *gyrB* were aligned by ClustalW (v.1.6), and Neighbour-joining trees were generated by 1000 bootstrap repetitions under the Kimura 2-parameter by MEGA X (Kumar et al., 2018). All positions containing alignment gaps and missing nucleotides were eliminated in pairwise sequence comparisons (the pairwise deletion option was used). The reference sequences were downloaded from the DDBJ/EMBL/GenBank database.

*Pulsed-field gel electrophoresis (PFGE)*

PFGE analysis was performed as described previously (Pei et al., 2008). Briefly, spirochetes cultured in BSK-H medium were harvested by centrifugation at 2,330 x g for 20 min at 4°C, washed three times with phosphate-buffered saline, and resuspended in 200 µl of distilled water. The concentration of bacterial cells was adjusted to 1 x 10^8^/300 µl. Samples were mixed with an equal volume of 1% SeaKem Gold Agarose (Lonza, ME, USA) to prepare the plugs. Bacterial cells were lysed in the plugs by incubation with lysis solution containing 1% proteinase K (FUJIFILM Wako Pure Chemical Corporation, Osaka, Japan) overnight at 50°C. The plugs were washed twice with TE buffer (pH 8.0, NIPPON GENE CO., LTD., Tokyo, Japan) containing 5 mM Pefabloc SC (Roche Diagnostics GmbH, Mannheim, Germany) at 50°C and stored in TE buffer at 4°C until use. PFGE was performed with the CHEF-DR III system (Bio-Rad Laboratories, Inc., CA, USA) at 6.0 V/cm with the pulse time ramped from 0.1 to 1.2 sec over 15 hrs. DNA bands were visualized by staining with ethidium bromide (Nakalai Tesque, Inc., Kyoto, Japan).

**References**

Chin CS, Alexander DH, Marks P, Klammer AA, Drake J, Heiner C, et al. Nonhybrid, finished microbial genome assemblies from long-read SMRT sequencing data. Nat Methods. 2013;10:563–9.

Kajitani R, Yoshimura D, Ogura Y, Gotoh Y, Hayashi T, Itoh T. Platanus_B: an accurate de novo assembler for bacterial genomes using an iterative error-removal process. DNA Res. 2020;27:dsaa014.

Kumar S, Stecher G, Li M, Knyaz C, Tamura K. MEGA X: Molecular evolutionary genetics analysis across computing platforms. Mol Biol Evol. 2018;35:1547–9.

Li H, Durbin R. Fast and accurate short read alignment with Burrows-Wheeler transform. Bioinformatics. 2009;25:1754–60.

Li H, Handsaker B, Wysoker A, Fennell T, Ruan J, Homer N, et al. The sequence alignment/map format and SAMtools. Bioinformatics. 2009;25:2078–9.

Pei Y, Terajima J, Saito Y, Suzuki R, Takai N, Izumiya H, et al. Molecular characterization of enterohemorrhagic *Escherichia coli* O157:H7 isolates dispersed across Japan by pulsed-field gel electrophoresis and multiple-locus variable-number tandem repeat analysis. Jpn J Infect Dis. 2008;61:58–64.

Walker BJ, Abeel T, Shea T, Priest M, Abouelliel A, Sakthikumar S, et al. Pilon: an integrated tool for comprehensive microbial variant detection and genome assembly improvement. PLoS One. 2014;9:e112963.
