# Supplementary figures and images for: The evolution of hard tick-borne relapsing fever borreliae is correlated with vector species rather than geographical distance
Source: BMC Ecol Evol. 2021 May 31;21:105. doi: 10.1186/s12862-021-01838-1 (PMC8166147; doi:10.1186/s12862-021-01838-1)

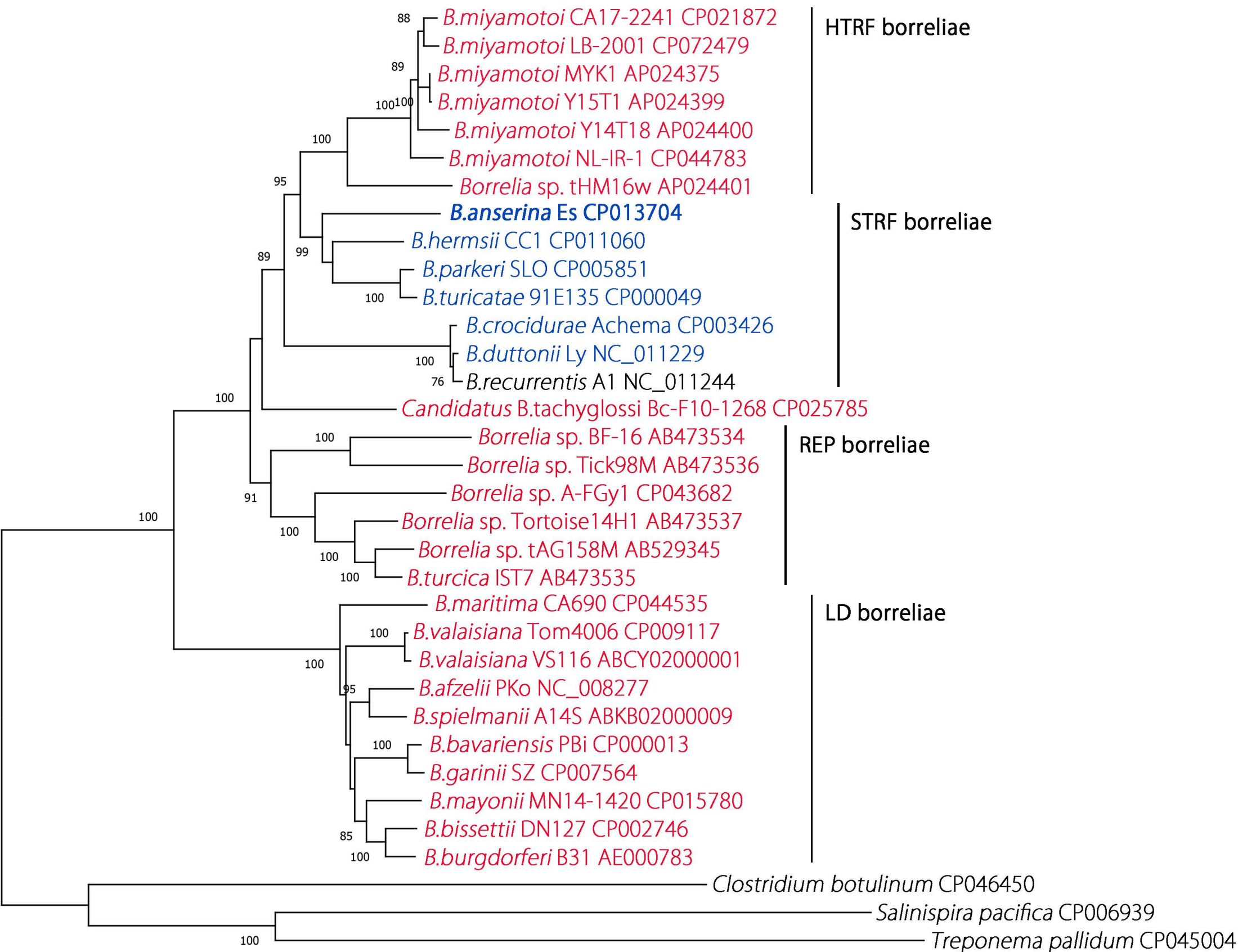

0.05

Supplement: Supplementary file 3 — Additional file 3: Figure S1. Phylogenetic trees of the entire borrelia lineage based on DNA gyrase subunit B gene (gyrB) gene. The phylogenetic tree is constructed by the neighbour-joining method based on the Kimura 2-parameter model. The percentage of replicate trees in which the associated taxa clustered together in the bootstrap test (1000 replicates) is indicated next to the branches. Values < 70% have been omitted. The bar indicates the percentage of sequence divergence. The bacteria transmitted by hard ticks, soft ticks, and lice are indicated in red, blue, and black, respectively. Sequence of B. anserina is indicated in bold type. GenBank accession numbers of each strains are indicated. [file 12862_2021_1838_MOESM3_ESM.pdf]

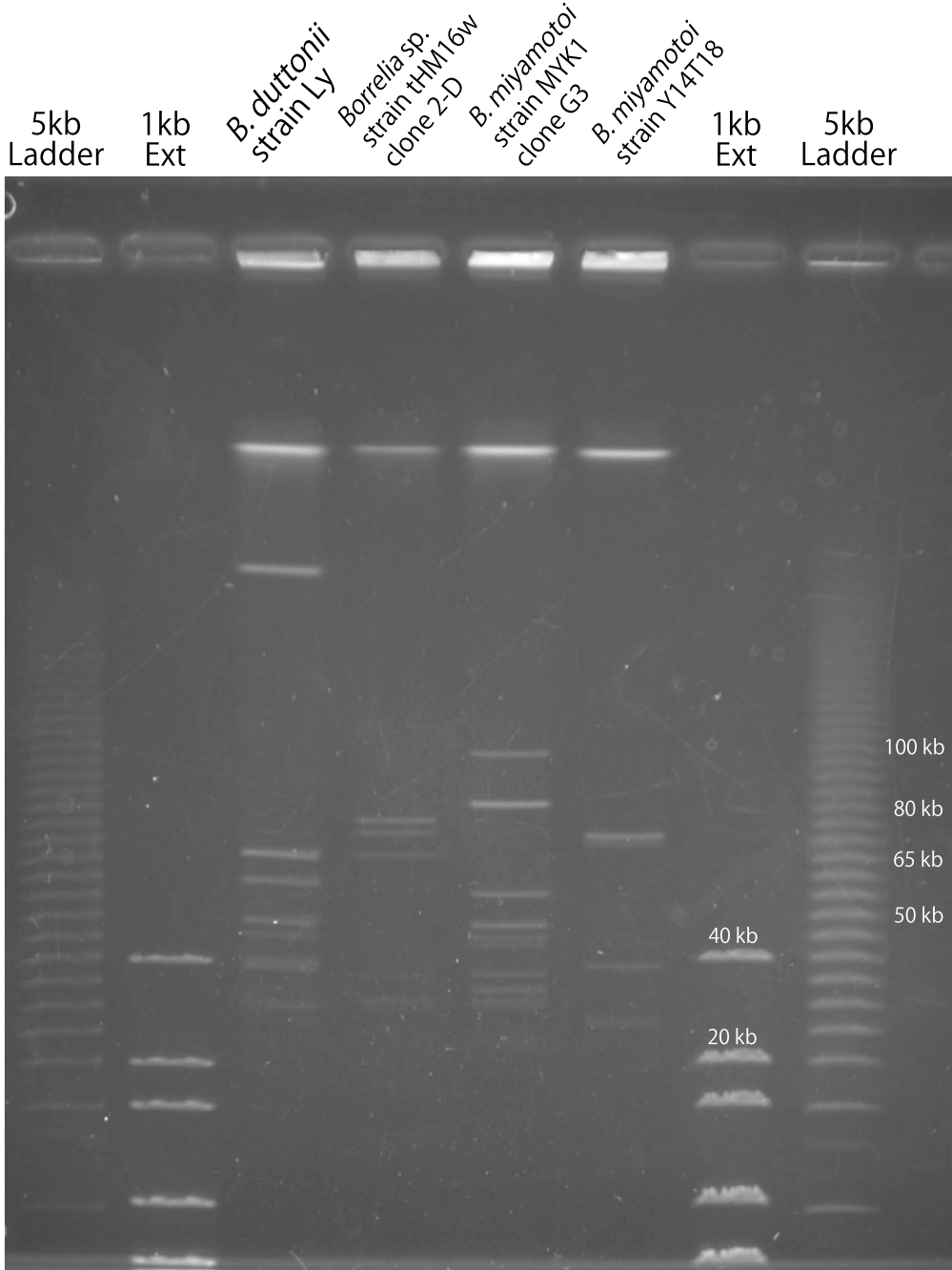

Supplement: Supplementary file 4 — Additional file 4: Figure S2. The plasmid repertoires of HTRF borreliae and STRF borreliae. [file 12862_2021_1838_MOESM4_ESM.pdf]

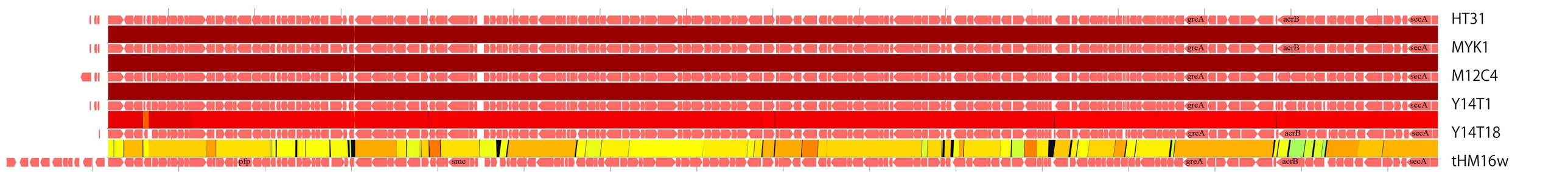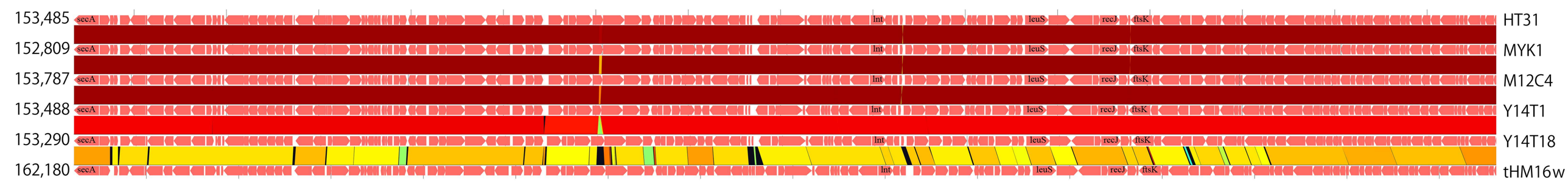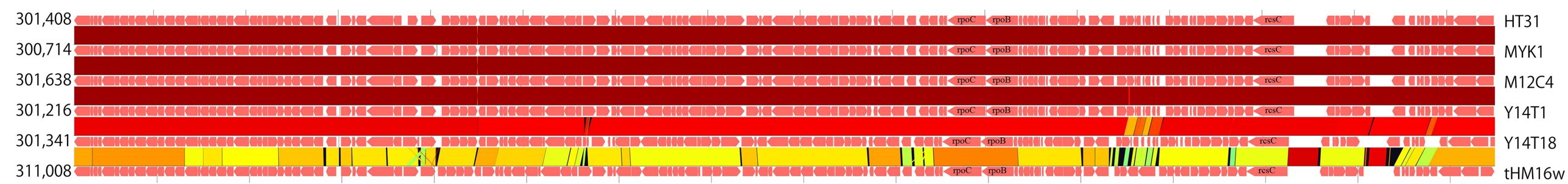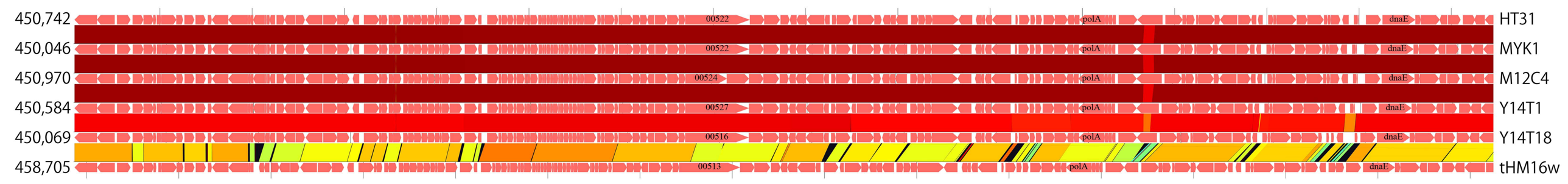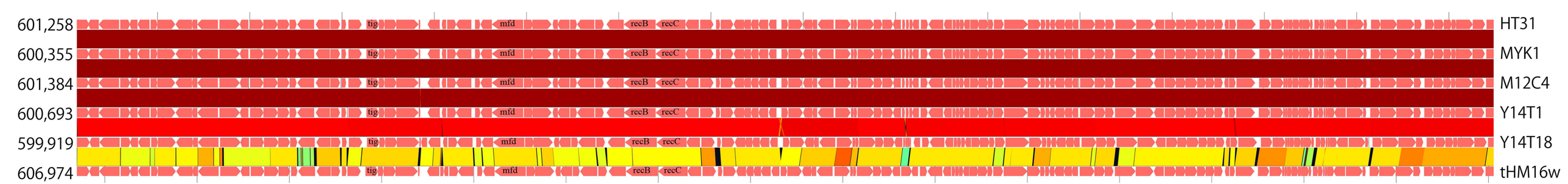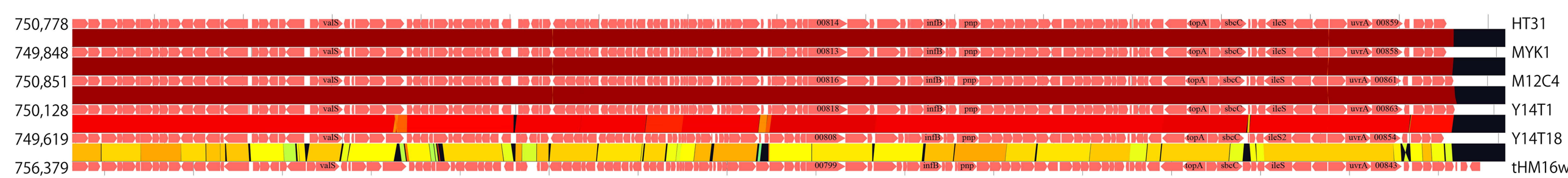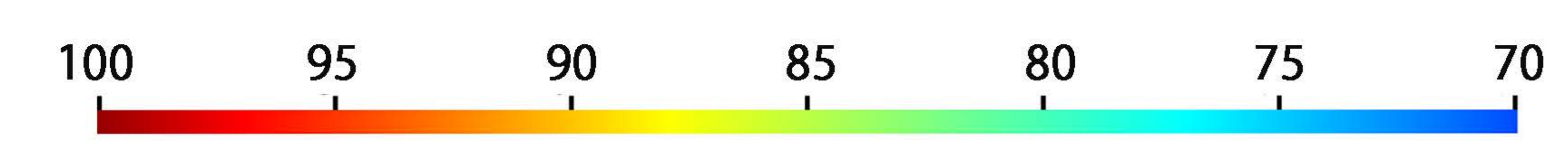

Supplement: Supplementary file 5 — Additional file 5: Figure S3. Comparison of the whole-chromosome sequences of the HTRF borreliae sequenced in this study. The entire chromosomal sequences of five B. miyamotoi strains (HT31, MYK1 G3, M12C4, Y14T1, and Y14T18) and Borrelia sp. tHM16w 2-D were compared using GenomeMatcher ver. 2.3. Sequence identity is shown by heat map. [file 12862_2021_1838_MOESM5_ESM.pdf]

BmHA00210

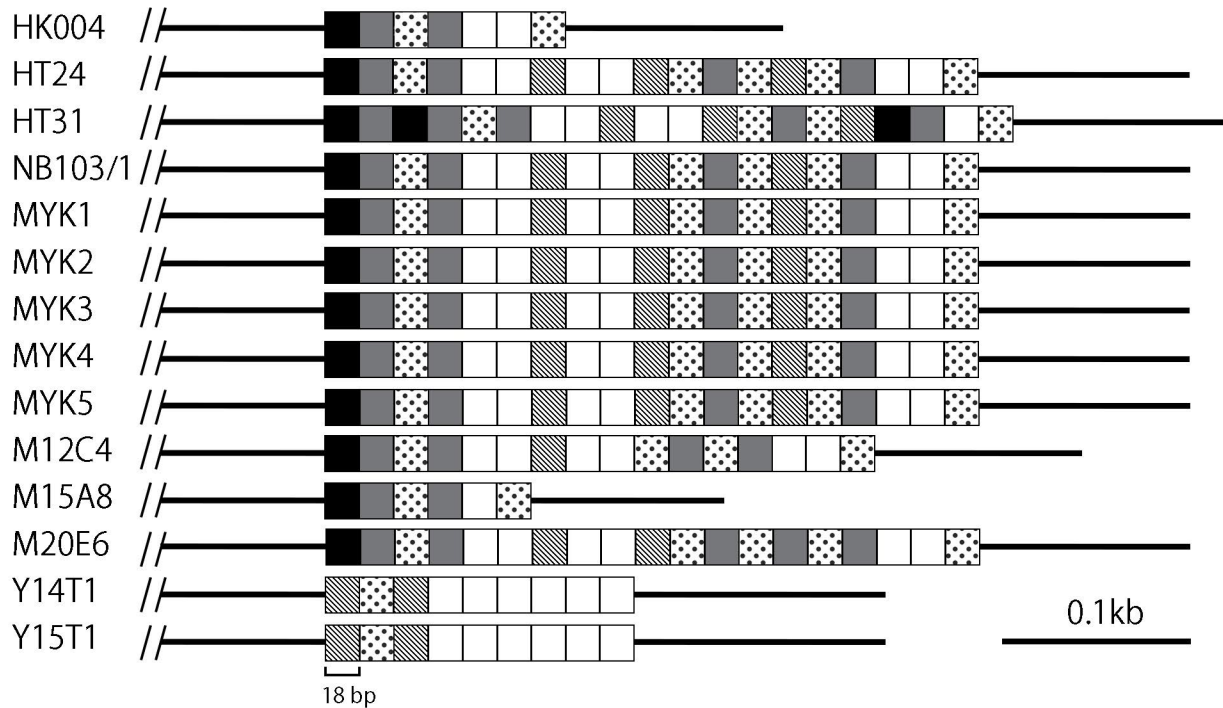

BmHA00402

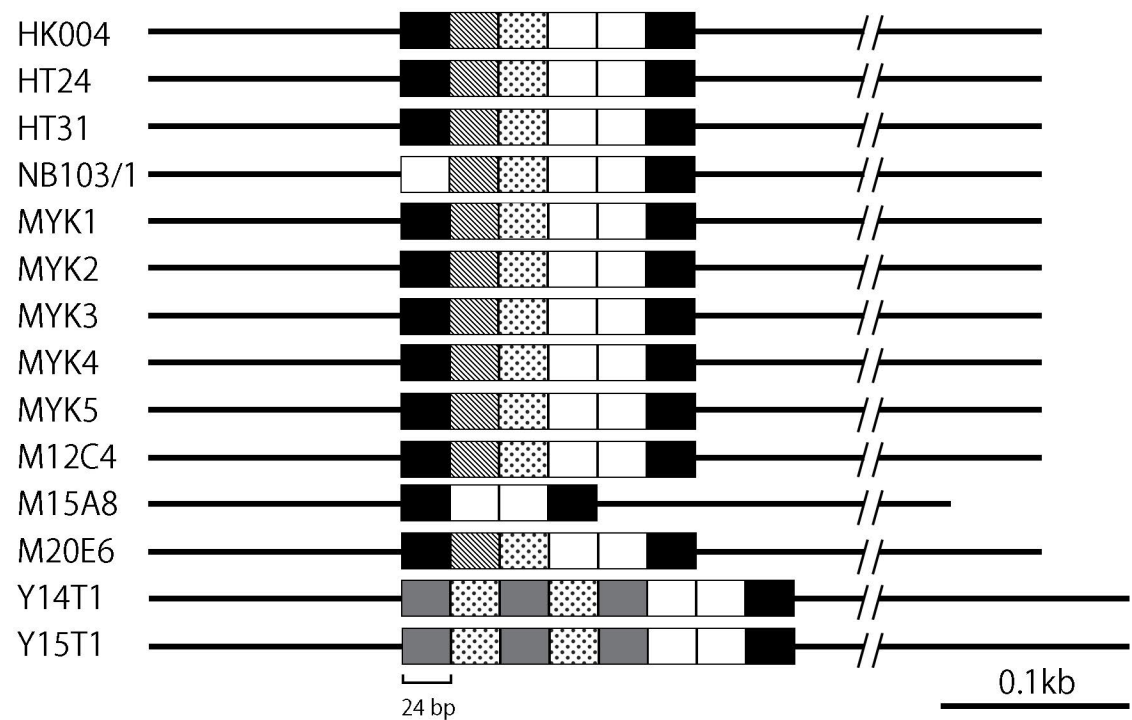

BmHA00563

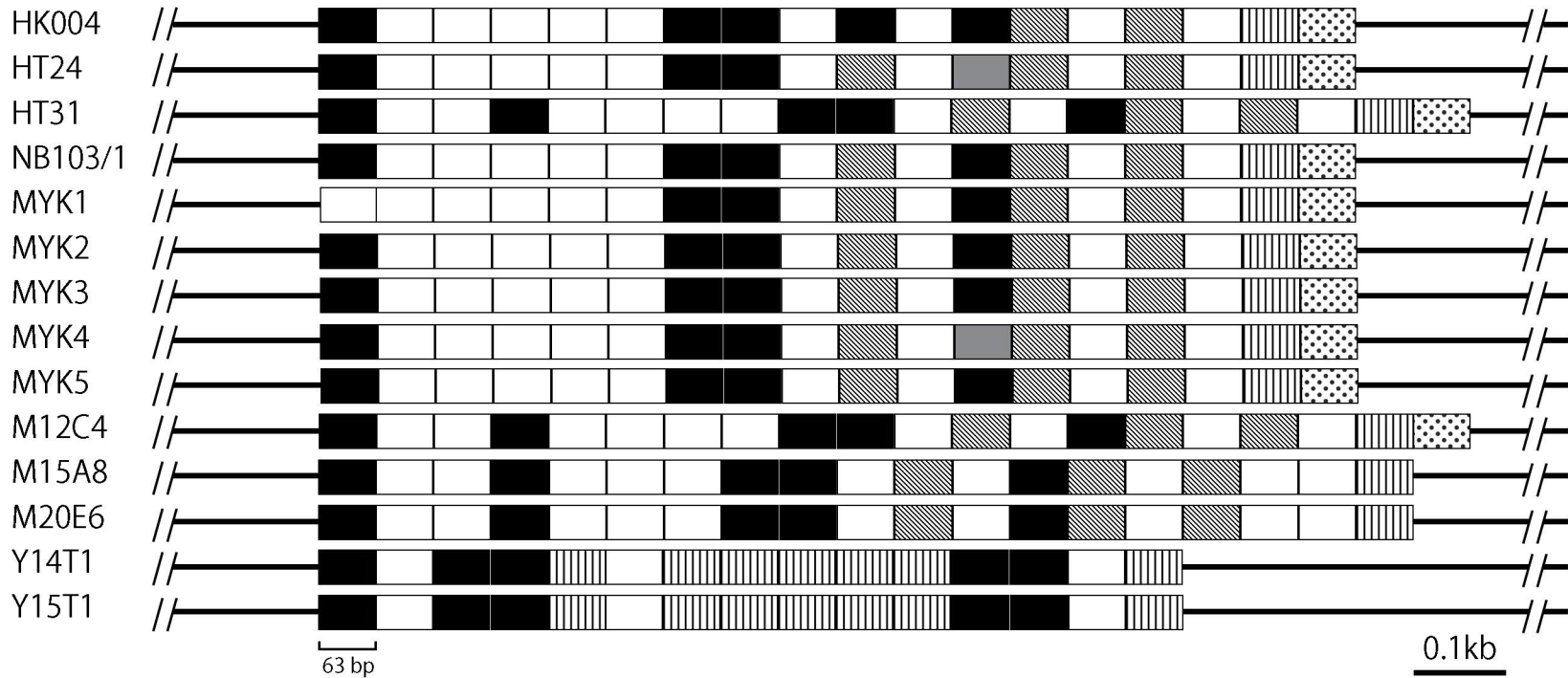

BmHA00703

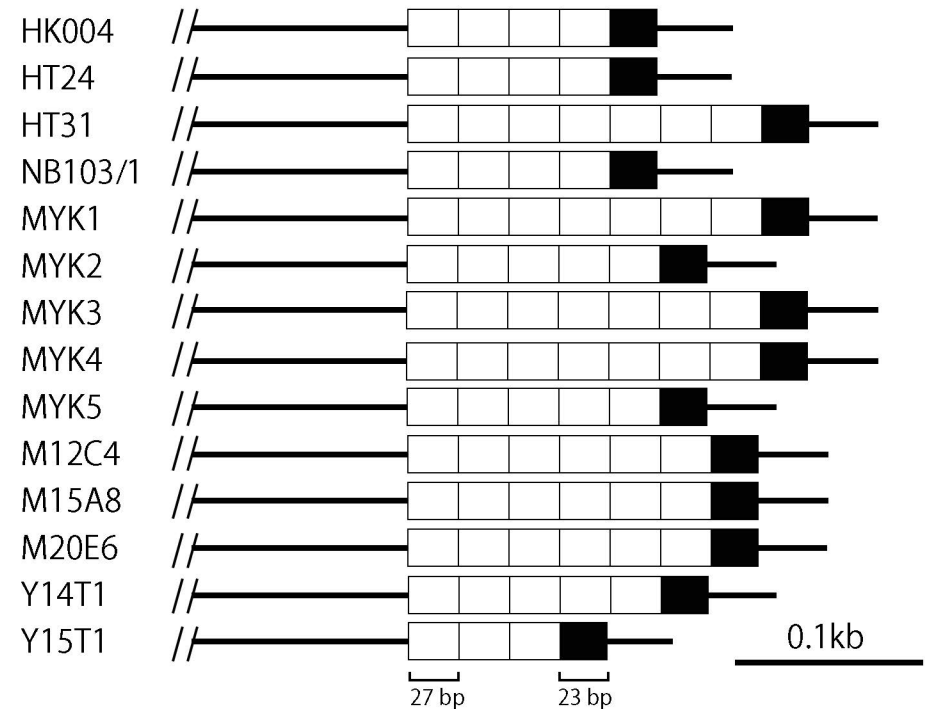

Supplement: Supplementary file 6 — Additional file 6: Figure S4. Schematic presentation of intragenic tandem repeats found in four B. miyamotoi genes. Tandem repeats in BmHA_00210, BmHA_00402, BmHA_00563 and BmHA_00703 are schematically shown. The tandem repeats were identified using Tandem Repeat Finder. Each box indicates a repeat unit, and boxes with the same colors and patterns represent the same or very similar repeat sequences (less than 4 base differences from the consensus sequence). [file 12862_2021_1838_MOESM6_ESM.pdf]
